# Anti‐Fibrotic MicroRNA‐Based Intervention in Oral Submucous Fibrosis: A Scoping Review

**DOI:** 10.1155/ijod/4230936

**Published:** 2026-08-27

**Authors:** Smitha Sammith Shetty, Mohit Sharma, Raghu Radhakrishnan

**Affiliations:** ^1^ Department of Oral and Maxillofacial Pathology and Microbiology, Manipal College of Dental Sciences, Manipal Academy of Higher Education, Manipal 576104, India, manipal.edu; ^2^ Department of Oral Pathology, Faculty of Dental Sciences, SGT University, Gurugram, Haryana, 122505, India, sgtuniversity.org; ^3^ Department of Oral and Maxillofacial Pathology, School of Clinical Dentistry, University of Sheffield, Sheffield S10 2TA, UK, sheffield.ac.uk; ^4^ Unit of Oral and Maxillofacial Pathology, Oman Dental College, Maabela South P.O. Box Mina Al Fahal, Muscat 116, Oman, odc.edu.om

**Keywords:** anti-fibrotic therapy, extracellular matrix, fibrosis, microRNA, oral submucous fibrosis, targeted therapy

## Abstract

**Background:**

Conventional therapies for oral submucous fibrosis (OSF) are largely conservative and have limited ability to modify the underlying fibrotic process or prevent disease progression. This limitation highlights the need for targeted molecular therapies. MicroRNAs (miRNAs) have recently emerged as promising anti‐fibrotic regulators capable of modulating key profibrotic signalling pathways.

**Objective:**

To systematically map and synthesise available evidence on antifibrotic miRNA‐based therapeutic interventions in OSF, including their mechanisms of action, delivery strategies, molecular targets and treatment outcomes.

**Methods:**

This scoping review followed the PRISMA‐ScR guidelines and employed the Arksey and O’Malley framework. A comprehensive search was performed in various databases such as PubMed, Scopus, Web of Science, Embase, Cochrane Library and Google Scholar to collect pertinent data. Studies considered eligible comprised of experimental, in vitro, in vivo, and quasi‐experimental research assessing anti‐fibrotic miRNAs in OSF models.

**Results:**

Of the 329 records identified, 10 studies fulfilled the inclusion criteria. Most employed in vitro fibrotic oral fibroblast models, with two incorporating in vivo animal models. Several miRNAs with antifibrotic properties were identified, including miR‐29b, miR‐760‐3p, miR‐145 inhibitor, miR‐424 inhibitor, miR‐503, miR‐30b‐5p, miR‐181a‐5p, miR‐29c, miR‐375 and miR‐204, which attenuate fibrotic responses by targeting genes and signalling pathways involved in fibrosis, including TGF‐β/Smad, RAS/RAF/MEK/ERK, and epithelial–mesenchymal transition (EMT).

**Conclusion:**

Anti‐fibrotic miRNA‐based interventions demonstrate promising antifibrotic activity in preclinical models of OSF by modulating the profibrotic pathways and reducing fibrosis. Advanced delivery platforms have helped to overcome challenges associated with miRNA delivery; however, evidence remains largely limited to in vitro and in vivo studies and does not establish reversal of established fibrosis or clinical efficacy in patients. Future research should prioritise standardisation of experimental models and well‐designed clinical trials for better understanding of therapeutic relevance and translational potential of miRNA in OSF.

## 1. Introduction

Oral submucous fibrosis (OSF) is a chronic and progressive condition that affects the oral mucosa, primarily in Southeast Asia, where the practice of chewing areca nut is widespread. This disorder is characterised by increasing stiffness of the mucosal tissue, a constant burning sensation, and a gradual reduction in the ability to open the mouth. These symptoms make eating and speaking more difficult, ultimately affecting the individual’s overall quality of life [1]. This condition is recognised as an oral potentially malignant disorder (OPMD) due to its established risk of transforming into oral cancer [2]. Epidemiological research indicates that the rate of malignant transformation varies between 7% and 13%, with more advanced stages of OSF showing a notably increased risk [1, 3, 4]. Pathologically, OSF is marked by an inflammatory reaction adjacent to the epithelium, leading to progressive fibro‐elastic alterations in the lamina propria and accompanied by epithelial atrophy [5]. This abnormal fibrotic process is driven by persistent inflammation, which maintains fibroblast activity, enhances collagen production, and hinders matrix degradation through the release of profibrotic cytokines, notably transforming growth factor‐β (TGF‐β) [6, 7]. The disturbance in the homeostatic equilibrium between collagen remodelling coupled with aberrant crosslinking promotes excessive collagen stabilisation and resistance to proteolytic breakdown, contributing to the progressive and largely irreversible nature of fibrosis observed in advanced OSF [7].

Recent advances in OSF treatment have transitioned from merely managing symptoms to adopting anti‐fibrotic methods that focus on the underlying mechanisms. These methods specifically target the fibrotic pathways involved in fibrosis, such as TGF‐β/Smad signalling, MEK1/ERK (mitogen‐activated protein kinase [MAPK]/extracellular signal‐regulated kinase) signalling, c‐Jun N‐terminal kinase (JNK), NF‐κB (nuclear factor kappa‐light‐chain‐enhancer of activated B cells) and p38 MAPK pathways [8]. Pharmacological strategies focus on altering the deposition of the extracellular matrix (ECM) by employing proteolytic enzymes like hyaluronidase [9] and collagenase [10]. They also aim to reduce chronic inflammation with the help of corticosteroids [9], interferon‐γ [11] and colchicine [12], while addressing microvascular compromise in OSF by using vasodilators such as isoxsuprine [13] and pentoxifylline [14]. Antioxidants and natural compounds like lycopene [15] and curcumin [16] mitigate OSF progression by counteracting oxidative stress [8]. Recent advancements indicate a paradigm shift towards targeting myofibroblast activation, immune dysregulation and epithelial–mesenchymal crosstalk. This is achieved with anti‐fibrotic therapies, which employ biomaterial‐based hydrogels (BG/HA) [17] and extracellular vesicles (EVs) sourced from mesenchymal stem cells [18].

With advances in molecular biology, microRNAs (miRNAs) have emerged as promising anti‐fibrotic agents given their capacity to regulate critical fibrogenic pathways including collagen production, ECM remodelling, and epithelial–mesenchymal transition (EMT) [19, 20]. Interestingly, certain miRNAs exert anti‐fibrotic effects by targeting ECM‐related genes, profibrotic mediators, or negatively regulating TGF‐β signalling, thereby attenuating fibrotic signalling cascades [20]. In this scoping review, we aimed to thoroughly explore the role of miRNA in OSF, with particular focus on anti‐fibrotic miRNA interventions, their mechanisms, therapeutic potential, and implications for future therapeutic research.

## 2. Methods

This scoping review was conducted and reported in accordance with the 2018 PRISMA‐ScR [21] guidelines (Supporting Information 1: Table S1), adhering to the methodological framework initially proposed by Arksey and O’Malley [22]. The review is registered with the Open Science Framework (OSF) under Registration DOI 10.17605/OSF.IO/YFS5E. The framework comprised the following steps.

### 2.1. Formulation of Research Questions

The research questions and sub‐questions were formulated as follows.

What are the different anti‐fibrotic miRNAs used in the treatment of OSF?

a. What are the different modes of treatment using anti‐fibrotic miRNAs in the treatment of OSF?
b. What are the outcomes of treatment with anti‐fibrotic miRNAs in OSF?

In this scoping review, the Population–Concept–Context (PCC) framework was utilised to explore questions regarding miRNA‐based anti‐fibrotic treatments for OSF.

#### 2.1.1. Population

The population of interest comprised cases of OSF across all stages of the disease. Studies conducted using animal models as well as in vitro experimental studies, were also included. Studies not related to OSF, those involving OSF with epithelial dysplasia, or studies focusing on other oral lesions were excluded.

#### 2.1.2. Concept

This review explored studies that investigated the use of anti‐fibrotic miRNAs as therapeutic interventions, including approaches that modulate miRNA activity through miRNA mimics/inhibitors or specialised delivery systems, and the resulting treatment outcomes. Studies focusing on pharmacological treatments not involving miRNAs, as well as those evaluating surgical interventions, were excluded.

#### 2.1.3. Context

Evidence from any clinical, laboratory, or translational research was considered without geographical restriction.

### 2.2. Study Designs

Studies addressing anti‐fibrotic miRNA interventions in OSF were included. This review encompassed both experimental and quasi‐experimental designs, such as randomised and non‐randomised controlled trials. Additionally, in vitro and in vivo studies, as well as systematic reviews meeting the inclusion criteria, were assessed for their relevance to the research question. Articles without available full texts were excluded, and only literature published in English was considered in this review.

### 2.3. Search Strategy and Identification of Relevant Literature

Electronic databases, including PubMed, Embase, Scopus, Web of Science, Cochrane Library and Google Scholar, were systematically searched to identify the grey literature. This search covered the period from the inception of these databases to the present, aiming to capture the latest advancements in miRNA research. The databases were searched on 29 December 2025 by the SSS and further validated by RR.

The search approach integrated both controlled vocabulary terms and free‐text keywords associated with OSF, fibrosis and miRNAs. Search strings were adapted for each database using appropriate indexing terms (e.g., MeSH and Emtree). The calibrated PubMed string used in the search was: ((((((oral submucous fibrosis[MeSH Terms]) OR (OSF[Title/Abstract])) OR (OSMF[Title/Abstract])) OR (“oral potentially malignant disorder”[Title/Abstract])) OR (“oral submucosal fibrosis”[Title/Abstract])) AND (((((microrna[MeSH Terms]) OR (MiRNA ^∗^[Title/Abstract])) OR (miR[Title/Abstract])) OR (“antifibrotic miRNA”[Title/Abstract])) OR (“antifibrotic miRNA”[Title/Abstract]))) AND ((intervention[Title/Abstract]) OR (therapy[Title/Abstract])) Supporting Information 2: Table S2.

### 2.4. Source of Evidence Selection

Following the search, the results were compiled and uploaded into Rayyan software [23], where duplicate entries were eliminated. A pilot test was conducted to evaluate the screening process. Subsequently, two reviewers, SSS and RR, independently assessed the titles and abstracts according to the inclusion criteria. Full texts of potentially relevant sources were obtained, and their citation details were imported into Rayyan software (Cambridge, MA 02142, USA) [23]. The selected citations underwent a comprehensive evaluation against the inclusion criteria by two independent reviewers, SSS and RR. Any discrepancies between the reviewers during the selection process were resolved through discussion or by consulting a third reviewer, MS.

### 2.5. Data Charting Methodology

A form for data collection was systematically designed and trialled to ensure that data extraction remained consistent across various study designs. Extracted data included bibliographic details, study design, model system (human, animal, in vitro), specific miRNAs investigated, treatment/delivery therapeutic strategy (miRNA mimic/inhibitor/delivery system), target genes, key signalling pathways, key outcomes related to fibrosis and reported limitations or confounders.

### 2.6. Synthesis of Results

The results were presented in a narrative format, with tables providing thematic support. The results were presented as authors, study year, country, the study design, the model (human oral mucosal fibroblasts [hOMFs], fibrotic buccal mucosa fibroblasts [fBMFs], rat or mouse model), anti‐fibrotic miRNAs, target genes, key pathways and key findings (Table 1).

**Table 1:** Characteristics of included studies.

Author (year)	Country	Study design	Cell line/model	miRNA / lncRNA	Treatment/delivery	Target gene(s)	Key pathway(s)	Biological outcome
Han et al. 2021 [18]	China	In vitro	fBMFs	miR‐375	ADSC‐derived EVs	FOXF1	Fibrotic signalling	miR‐375 inhibited the transition of fibroblast to myofibroblasts, decreased proliferation, migration, and invasion, elevated apoptosis of fBMFs, and inhibited levels of fibrosis markers α‐SMA, collagen I, collagen III
Cheng et al. 2025 [24]	Taiwan	In vitro	hOMFs	miR‐145 miR‐424	Chitosan nanoparticles with miR‐145 and miR‐424 inhibitors	TGFB1, ACTA2, COL1A, COL3A, COL4A, TIMP2, and ZEB1	TGF‐β/Smad signalling	Chitosan nanoparticle‐based miRNA delivery reduced fibroblast activation and collagen synthesis exhibiting strong antifibrotic activity.
Yang et al. 2022 [25]	Taiwan	In vitro	fBMFs	miR‐29c	miRNA mimic	Tropomyosin‐1 (TPM1)	TGF‐β/Smad signalling	miR‐29c reduced migration ability and collagen gel contractility of fBMFs, thus demonstrating an inhibitory effect on myofibroblast activation
Yu et al. 2021 [26]	Taiwan	In vitro	fBMFs from OSF tissues, 293T cells	miR‐29b H19	miR‐29b mimic + silencing of H19 via shRNA	COL1A1 ACTA2 fibronectin	TGF‐β/Smad3 signalling	miR‐29b reversed fibrosis by reducing type 1 collagen and myofibroblast activities
Lee et al. 2021 [27]	Taiwan	In vitro	fBMFs	miR‐204 LINC00084	miRNA mimic (+silencing of LINC00084)	ZEB1	EMT	miR‐204 decreases the expression of the epithelial‐mesenchymal transition inducer ZEB1, thereby abrogating the LINC00084‐induced myofibroblast activation.
Fu et al. 2025 [28]	China	In vitro + In vivo	hOMFs; OSF Rat models	miR‐29b	Tetrahedral framework nucleic acids (tFNAs) Nanodrug	COL1A1, ACTA2, and fibronectinSmad2/3	TGF‐β/Smad signalling pathway, Nrf2/HO‐1 antioxidant pathway,	T‐29b (tFNA+miR‐29b) inhibited the activation of human oral mucosal fibroblasts (hOMF) and reduced the expression of fibrosis‐related proteins like α‐SMA and Collagen I
Wang et al. 2023 [29]	China	In vitro + In vivo	fBMFs; OSF mouse model	miR‐760‐3p	ADSC‐derived EVs	IGF1R	TGF‐β1/Smad3	EV‐mediated miR‐760‐3p suppress cell proliferation, migration, and invasion, and reduce levels of fibrosis markers α‐SMA, collagen I, and collagen III in fBMFs.
Wen et al. 2024 [30]	China	In vitro	hOMFs	miR‐503	miRNA mimic	RAF	RAS/RAF/MEK/ERK	miR‐503 inhibits the proliferation, migration, differentiation, and collagen synthesis of PDGF‐BB‐induced oral mucosal FBs.
Xie et al. 2024 [31]	China	In vitro	hOMFs	miR‐30b‐5p	Puerarin treatment and miRNA mimic	FAP	Fibroblast activation	Natural compound‐induced miR‐30b‐5p suppressed the excessive proliferation of hOMFs and declined fibrosis with downregulation of COL3A1, COL1A1, MMP1 and MMP3
Shao et al. 2024 [32]	China	In vitro	fBMFs	miR‐181a‐5p	ADSC‐derived exosomes	Smad2	TGF‐β/Smad	miR181a‐5p suppressed myofibroblast transdifferentiation.

### 2.7. Quality Assessment of Studies

The ToxRTool criteria, known as the Toxicological Data Reliability Assessment Tool [33], was employed for methodological quality assessment of studies included in the Scoping review. Although originally developed for toxicological research, ToxRTool provides a structured and transparent framework for evaluating key methodological domains, including study design, characterisation of the test system, description of experimental procedures, reporting of outcomes, and documentation of results for both in vitro and in vivo experiments [33]. These criteria are equally relevant to in vitro and in vivo miRNA studies, where experimental design and reproducible reporting are essential for interpreting biological effects. Thus, ToxRTool for in vivo studies evaluated the methodology using a 21‐point checklist that covers aspects such as the test substance and test organism identification, the study design, the results, and the plausibility of both the design and data. Studies scoring between 18 and 21 points are deemed reliable without restrictions, those scoring 13–17 points are considered reliable but with potential restrictions, and those with fewer than 13 points are regarded as unreliable. In the case of in vitro studies, the tool uses an 18‐point checklist focusing on the test substance and system identification, the study design, the results, and the plausibility of the design and data. Studies achieving 15–18 points are considered reliable without restrictions, those with 11–14 points are reliable with possible restrictions, and studies scoring below 11 points are deemed unreliable [33].

## 3. Results

**Figure 1:**
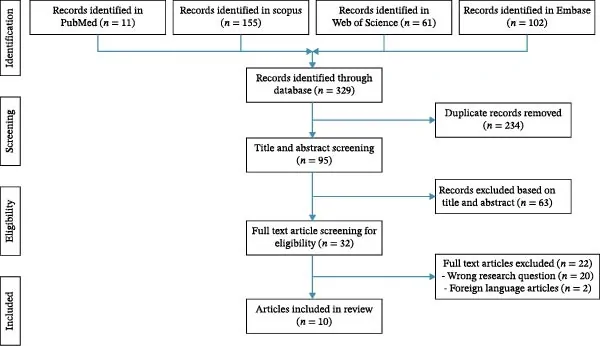
A PRISMA flowchart of the study selection process.

Searches were electronically performed on several databases: PubMed (MEDLINE) (*n* = 11), Scopus (*n* = 155), Web of Science (Clarivate) (*n* = 61), EMBASE (Elsevier) (*n* = 102), Cochrane (*n* = 0) and Google Scholar, with no relevant grey literature found. Out of the 329 records obtained, 234 duplicates were eliminated using Rayyan. Subsequently, 95 articles underwent Title Abstract screening, and 32 were deemed suitable for full‐text review. From these 32 articles, 10 were selected for analysis, while the rest were excluded due to incorrect research questions (*n* = 20) and articles in foreign languages (*n* = 2). Figure 1 displays the PRISMA flow diagram.

### 3.1. Characteristics of Included Studies

#### 3.1.1. Study Settings and Study Design

Among the included studies, four studies were conducted in Taiwan [24–27] and six of the studies in China [18, 28–32]. All included studies predominantly employed in vitro models to explore the anti‐fibrotic mechanism of miRNAs in OSF at the cellular level to elucidate regulating molecular pathways, miRNA–target gene interactions, and regulation of downstream fibrotic markers. Two studies incorporated in vivo OSF animal models, which complemented the in vitro findings and helped validate their translational relevance [28, 29] (Table 1).

#### 3.1.2. Experimental Models and Interventions

Most studies utilised arecoline or TGF‐β‐induced fibrotic oral fibroblast models, including hOMFs [24, 28, 30, 31] and fBMFs [18, 25–27, 29, 32], to mimic the OSF associated fibrosis. These models consistently demonstrated an increase in collagen deposition, myofibroblast differentiation and upregulation of fibrotic markers, thereby providing a robust platform for evaluating anti‐fibrotic interventions.

Therapeutic interventions primarily focused on miRNA modulation to counteract fibrotic signalling. The different approaches included the use of miRNA mimics/inhibitors [24–27, 30], EVs [18, 29, 32] or nanoparticle (NP)‐based miRNA delivery systems, which are designed to enhance miRNA stability and cellular uptake [24, 28]. In addition, two studies investigated the natural compounds as upstream regulators of anti‐fibrotic miRNAs [24, 31], highlighting the potential of phytochemicals to indirectly modulate fibrotic pathways through miRNA‐mediated mechanisms (Table 1).

#### 3.1.3. Anti‐Fibrotic miRNAs

##### 3.1.3.1. miRNAs Exhibiting Anti‐Fibrotic Mechanistic Role in OSF

A diverse set of miRNAs demonstrated anti‐fibrotic activity in OSF models, highlighting the complexity and redundancy of miRNA‐mediated regulation of fibrosis. The anti‐fibrotic miRNAs identified from the reviewed studies that modulate the molecular pathways involved in OSF‐associated fibrosis are miR‐29c [25], miR‐29b [26], miR‐204 [27] and miR‐503 [30].

##### 3.1.3.2. miRNAs With Anti‐Fibrotic Therapeutic Potential in OSF

miRNAs hold considerable promise as therapeutic agents for OSF, their application is hindered by challenges such as instability, inefficient cellular uptake and delivery‐specific limitations. The reviewed studies reported the successful delivery of miRNAs, including miR‐375 [18], miR‐145 inhibitor, miR‐424 inhibitor [24], miR‐29b [28], miR‐760‐3p [29], miR‐30b‐5p [31] and miR‐181a‐5p [32] using advanced delivery platforms.

##### 3.1.3.3. miRNA Targets and Regulating Pathways

**Figure 2:**
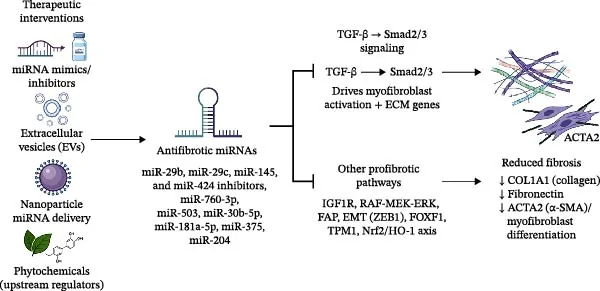
miRNA‐based anti‐fibrotic interventions in OSF.

The anti‐fibrotic miRNAs identified exhibit their anti‐fibrotic role by targeting genes that drive increased collagen production, promote myofibroblast differentiation, or regulate key signalling pathways involved in fibrosis. miR‐29b emerged as a central regulator, targeting COL1A1, ACTA2, fibronectin, downstream of the Nrf2/HO‐1 antioxidant pathway and TGF‐β/Smad2/3 pathway [26, 28]. Similarly, miR‐145 and miR‐424 inhibitors targeted TGF‐β1, COL1A1 and ACTA2, leading to repression of the TGF‐β/Smad pathway [24]. miR‐760‐3p exerted anti‐fibrotic effects by targeting insulin‐like growth factor 1 receptor (IGF1R), subsequently inhibiting TGF‐β1/Smad3 signalling, while miR‐181a‐5p directly suppressed Smad2, further dampening TGF‐β–driven fibrogenesis [29]. miR‐503 targets RAF and modulates the RAS/RAF/MEK/ERK pathway [30]. In addition, miR‐30b‐5p inhibited fibroblast activation protein (FAP), reducing fibroblast activation [31]. miR‐29c targeted tropomyosin‐1 (TPM1) [25], leading to suppression of TGF‐β/Smad signalling, while miR‐375 inhibited FOXF1 [18], a transcription factor associated with fibrotic signalling pathways. Furthermore, miR‐204 targeted ZEB1 [27], resulting in inhibition of EMT, a process that contributes to fibroblast accumulation and fibrosis (Figure 2) [34].

#### 3.1.4. Quality Assessment

Among the 10 studies evaluated, two conducted both in vivo and in vitro experiments and were classified as ‘reliable without restriction’ for both types. Of the eight studies that exclusively focused on in vitro experiments, six were rated as ‘reliable without restriction’, while two were categorised as ‘reliable with restriction’ based on the ToxRTool criteria. Overall, the studies included in the review exhibited strong methodological quality. The evaluation of the methodological quality of the studies included in the scoping review is detailed in Table 2. The quality assessment of the included studies was undertaken to characterise methodological reliability and reporting quality rather than to interpret the risk of bias, consistent with the exploratory objectives of a scoping review.

**Table 2:** Quality assessment of studies using ToxRTool criteria.

In vivo studies Author/year of publication	Group I: Test substance identification	Group II: Test organism characterisation	Group III: Study design description	Group IV: Study results documentation	Group V: Plausibility of study design and results	Total	Reliability categorisation
Fu et al. 2025 [28]	3	3	7	3	2	18	Reliable without restrictions
Wang et al. 2023 [29]	3	5	6	3	2	19	Reliable without restrictions

**In vitro Studies** **Author/ Year of publication**	**Group I: Test substance identification**	**Group II: Test system characterisation**	**Criteria Group III: Study design description**	**Criteria Group IV: Study results documentation**	**Criteria Group V: Plausibility of study design and data**	**Total**	**Reliability categorisation**

Han et al. 2021 [18]	3	3	4	3	2	15	Reliable without restrictions
Cheng et al. 2025 [24]	3	3	5	3	2	16	Reliable without restrictions
Yang et al. 2022 [25]	2	3	2	3	2	12	Reliable with restrictions
Yu et al. 2021 [26]	2	3	5	3	2	15	Reliable without restrictions
Lee et al. 2021 [27]	2	3	5	3	2	15	Reliable without restrictions
Fu et al. 2025 [28]	3	3	5	3	2	15	Reliable without restrictions
Wang et al. 2023 [29]	3	3	5	3	2	15	Reliable without restrictions
Wen et al. 2024 [30]	3	2	4	3	2	14	Reliable with restrictions
Xie et al. 2024 [31]	3	3	5	3	2	15	Reliable without restrictions
Shao et al. 2024 [32]	3	3	5	3	2	16	Reliable without restrictions

## 4. Discussion

OSF exhibits the potential for malignant change with progression and is characterised by a gradual and progressive fibrotic transformation of the submucosal connective tissue, leading to functional and structural impairment of the oral mucosa [35]. This review seeks to systematically document effective anti‐fibrotic miRNA therapeutic interventions for OSF, highlighting existing gaps in management and emphasising the urgent need for efficient treatment strategies for the reversal of fibrosis.

### 4.1. Targeted Anti‐Fibrotic Therapy Using NP‐Based miRNA Delivery Systems

NP‐based drug delivery systems present a favourable strategy to facilitate targeted and site‐specific drug delivery and allowing sustained therapeutic effects [36]. Among the diverse nano‐sized carriers explored for therapeutic applications in fibrosis, glycochitosan NPs (CNPs), derived from chitosan (CS), have gained considerable attention as a promising delivery platform owing to their capacity to efficiently encapsulate siRNAs, miRNAs and other therapeutic agents, making them suitable for targeted gene‐based interventions in fibrosis [37]. Similarly, tetrahedral framework nucleic acids (tFNAs) are a novel type of three‐dimensional DNA nanomaterial, formed by assembling four single‐stranded DNA (ssDNA) strands that are precisely designed [38]. They act as efficient carriers for small‐molecule drugs and miRNAs, making them a promising platform for intracellular drug delivery. As a result, tFNAs can function as effective vehicles for transporting miRNAs into cells, enabling them to exert their intended biological activities [39].

In this review, we identified two studies that investigated the efficiency of NP‐based miRNA interventions in OSF. Cheng et al. (2025) proposed CS NPs as an efficient delivery system for miRNAs, including miR‐145 and miR‐424 [24]. These nanoencapsulations shield the negatively charged groups in miRNAs to facilitate better cellular uptake and improve the delivery efficiency. CS‐mediated delivery of miR‐145 and miR‐424 inhibitors significantly reduced fibroblast activation and collagen synthesis, demonstrating potent anti‐fibrotic effects [24]. This effect was mediated through the downregulation of mRNA expression of significant profibrotic markers, namely, TGFB1, TIMP2, COL1A1, COL3A1, COL4A1, ACTA2 and ZEB1, in arecoline‐stimulated myofibroblasts, thereby suppressing the profibrotic TGF‐β signalling pathway. These results suggest that miRNA‐loaded CS NPs (miR/CS NPs) have the potential to enhance the delivery efficiency in the treatment of OSF [24]. Fu et al. [28] (2025) investigated the anti‐fibrotic effects of miR‐29b in OSF through a new nanodrug named T‐29b, which consists of tFNAs carrying miR‐29b. Studies conducted in vitro and on arecoline‐induced OSF rat models revealed that T‐29b significantly inhibits the activation of hOMFs and decreases the levels of fibrosis‐associated proteins like α‐SMA and collagen I. Additionally, T‐29b was observed to lower the generation of reactive oxygen species (ROS) in hOMFs by influencing the TGF‐β/Smad and Nrf2/HO‐1 signalling pathways [28]. Arecoline‐induced OSF models showed improved epithelial and lamina propria morphology with reduced collagen deposition following T‐29b treatment, suggesting its potential therapeutic utility in OSF treatment [28].

### 4.2. Adipose Tissue‐Derived Stromal Cell (ADSC)‐Derived EVs/Exosomes–Mediated Anti‐Fibrotic miRNA Therapy

Adipose tissue‐derived stromal cells (ADSCs), which are a distinct group of mesenchymal stromal cells sourced from adipose tissue, release EVs crucial for communication between cells. These EVs function as biological transporters, carrying miRNAs, proteins, and cytokines, thus enabling cell signalling and the exchange of regulatory molecules among cells [29]. We reviewed three studies that investigated the specific roles and therapeutic efficacy of ADSC‐derived EVs in reducing fibrosis. Shao et al. [32 studied the anti‐fibrotic potential of miR‐181a‐5p‐enriched ADSC‐Exosomes (ADSC‐Exo) on OSF. The study reported that treatment of arecoline‐induced myofibroblasts with ADSC‐Exo promoted cellular proliferation and migration but attenuated myofibroblast differentiation and significantly inhibited collagen deposition. The combined effects resulted in an anti‐fibrotic outcome, which in turn inhibited the progression of OSF [32]. miR‐181a‐5p, which is abundantly expressed in ADSC‐Exo, directly binds to the 3^′^‐UTR of the Smad2 mRNA. This binding inhibits the TGF‐β signalling pathway, thereby exerting anti‐fibrotic effect OSF [32]. Wang et al. [29] explored the potential of EVs derived from ADSC‐EVs to reduce OSF by influencing the expression of the anti‐fibrotic miR‐760–3p. Their study demonstrated that ADSC‐EVs significantly upregulated miR‐760‐3p, leading to reduced cell proliferation, migration and invasion, along with decreased expression of fibrosis‐associated markers in fBMFs [29]. The anti‐fibrotic effects of ADSC‐EVs were mediated via the miR‐760‐3p/IGF1R axis, which subsequently inhibited the activation of the TGF‐β1/Smad3 signalling pathway [29]. The OSF model treated with ADSC‐EVs exhibited improved epithelial and lamina propria architecture compared with the ADSC‐EV group with miR‐760‐3p inhibition, which demonstrated buccal mucosal epithelial contraction, increased inflammatory cell infiltration and reduced vascularity within the lamina propria, highlighting the role of miR‐760‐3p in mediating the therapeutic effects of ADSC‐EVs [29]. Similarly, Han et al. (2021) investigated the potential role of ADSC‐EVs transduced with miR‐375 on OSF‐associated fBMFs. Overexpression of miR‐375 in ADSC‐EVs significantly reduced invasion, proliferation and migration while promoting apoptosis of fBMFs through suppression of its target gene FOXF1 [18]. Consequently, ADSC‐EVs exerted anti‐fibrotic effects and attenuated the progression of OSF via modulation of the miR‐375/FOXF1 signalling axis. This is further supported by the downregulation of key fibrosis‐associated markers, including collagen type I/III and α‐SMA [18].

### 4.3. miRNA Mimic–Driven Strategies to Elucidate Anti‐Fibrotic Mechanisms

miRNA‐based anti‐fibrotic therapies are carried out by upregulating specific miRNAs, which can be achieved either through the administration of synthetic miRNA mimics or via the delivery of vectors engineered to express the desired miRNAs [40]. Several studies employed miRNA mimics to investigate the anti‐fibrotic potential of miRNAs. Wen et al. [30] investigated the anti‐fibrotic potential of miR‐503 in OSF and its interaction with platelet‐derived growth factor‐BB (PDGF‐BB). PDGF‐BB is recognised as a potent profibrotic mediator that stimulates the proliferation of oral mucosal fibroblasts and facilitates their transformation into myofibroblasts, thereby promoting fibrosis [41]. The overexpression of miR‐503, facilitated through miRNA mimics, effectively suppressed PDGF‐BB‐induced differentiation, proliferation, migration and collagen synthesis in oral mucosal fibroblasts. This suppression was evidenced by the lower expression of fibrotic markers, including collagen‐I and α‐SMA [30]. miR‐503 achieves this protective effect by negatively regulating RAF expression, thereby inhibiting the RAS/RAF/MEK/ERK signalling pathway. By attenuating this pathway, miR‐503 effectively curtails the profibrotic response initiated by PDGF‐BB in oral mucosal fibroblasts, underscoring its potential as an anti‐fibrotic therapeutic agent in OSF [30]. Yu et al. [26] reported that the long non‐coding RNA H19 acts as a competitive endogenous RNA (ceRNA) that binds and sequesters the anti‐fibrotic miRNA miR‐29b, thereby blocking its ability to regulate type I collagen (COL1A1). Silencing H19 increases miR‐29b expression in fBMFs, which subsequently reduces myofibroblast‐like characteristics [26]. Experimentally increasing miR‐29b levels in fBMFs through miRNA mimics results in reduced collagen gel contraction and cell migration. This intervention also diminishes the expression of critical fibrosis markers, including α‐SMA, type I collagen, and fibronectin, thereby exerting an anti‐fibrotic effect [26]. Yang et al. [25] documented that miR‐29c expression was decreased in fBMFs obtained from OSF specimens. By experimentally increasing the expression of miR‐29c through miRNA mimics, a decrease in cell migration and collagen gel contractility was observed, suggesting that miR‐29c inhibits the activation of myofibroblasts [25]. Tropomyosin‐1 (TPM1), a cytoskeletal regulatory protein, has been identified as a direct target of miR‐29c. TPM1 plays a crucial role in stabilising actin filaments and facilitating actin–myosin interactions, which are essential for cell motility and myofibroblast activity [42]. TPM1 is upregulated in TGF‐β/Smad signalling and facilitates the activation of fibrotic cells. Thus, miR‐29c‐mediated suppression of TPM1 limits cytoskeletal remodelling, reduces myofibroblast activation, and attenuates fibrosis [25]. Xie et al. [31] investigated the anti‐fibrotic effects of the Chinese medicine Puerarin (Pue) in OSF. Pue intervention effectively reduced the overproduction of hOMFs triggered by TGF‐β1 and restore the expression levels of the anti‐fibrotic miR‐30 family, with miR‐30b‐5p showing the most notable change [31]. Thus, experimental transfection of hOMFs with a miR‐30b‐5p mimic suppressed TGF‐β1–induced cell proliferation. Additionally, miR‐30b‐5p suppressed the TGF‐β1‐induced expression of FAP and its downstream fibrotic targets, such as MMP1, MMP3, COL1A1, and COL3A1, thus demonstrating a substantial anti‐fibrotic effect [31]. Lee et al. [27] reported that the long non‐coding RNA LINC00084 is significantly upregulated in OSF tissues and in fBMFs. Mechanistically, LINC00084 functions as a molecular sponge for miR‐204, thereby elevating the expression of the transcription factor ZEB1, which promotes myofibroblast differentiation while suppressing the anti‐fibrotic activity of miR‐204 [27]. Conversely, silencing LINC00084 increases miR‐204 levels in fBMFs, leading to reduced myofibroblast activation via the downregulation of ZEB1 [27]. Experimental overexpression of miR‐204 with miR‐204 mimics showed that both collagen gel contractility and migration were decreased in fBMFs, exhibiting an anti‐fibrotic role in OSF [27].

This scoping review is limited by the scarcity of evidence pertaining to the anti‐fibrotic role of miRNAs, which is predominantly derived from in vitro and animal studies. All the included studies are from China or Taiwan; this limits the generalizability of the evidence to other populations due to the variability in genetic background, environmental exposures, and different forms of areca nut consumption. Further, the current miRNA‐based interventions demonstrate the modulation of fibrotic pathways and repression of fibrotic factors resulting in decreased collagen deposition, but whether these approaches can reverse established fibrosis remains unclear.

## 5. Conclusion

This scoping review provides insights into current preclinical evidence on the anti‐fibrotic potential of miRNA‐based interventions in OSF. The available experimental studies indicate that specific miRNAs can effectively suppress fibroblast activation, collagen accumulation, and profibrotic signalling pathways, particularly those mediated by TGF‐β. Advanced delivery platforms such as NPs, EVs, and nucleic acid nanostructures further enhance therapeutic stability, targeting and cellular uptake. However, the current evidence base is largely limited to in vitro studies, with minimal in vivo validation and no clinical trials of OSF to date. Consequently, the current evidence supports the attenuation or prevention of fibrotic changes in experimental models rather than the reversal of established fibrosis or clinical efficacy in humans. Future research should prioritise standardisation of experimental models, longitudinal animal studies, evaluating long‐term safety and well‐designed clinical trials to determine therapeutic efficacy and feasibility of miRNAs‐based therapies. Collectively, the available evidence suggests that anti‐fibrotic miRNAs may represent a potential therapeutic approach for modulating fibrosis‐related pathways in OSF; however, their clinical applicability remains uncertain and requires further preclinical and clinical validation.

## Author Contributions

**Smitha Sammith Shetty:** conceptualisation, methodology, software, data curation, investigation, validation, formal analysis, visualisation, writing – original draft. **Mohit Sharma:** methodology, investigation, data curation, writing – reviewing and editing. **Raghu Radhakrishnan**: conceptualisation, methodology, software, investigation, supervision, project administration, writing – review and editing.

## Funding

This study did not receive any specific funding.

## Disclosure

All authors have read and approved the final version of the manuscript. Raghu Radhakrishnan: corresponding author, had full access to all the data in this study and takes complete responsibility for the integrity of the data and the accuracy of the data analysis.

## Ethics Statement

Ethical approval was not required for this study as it is a scoping review.

## Conflicts of Interest

The authors declare no conflicts of interest.

## Supporting Information

Additional supporting information can be found online in the Supporting Information section.

## Supporting information


**Supporting Information 1** Table S1: PRISMA‐ScR Checklist for Scoping Reviews.

**Supporting Information 2** Table S2: Search Strategy.

## Data Availability

Data sharing not applicable—no new data generated or the article describes entirely theoretical research.
