# Supplementary material for: Anti‐Fibrotic MicroRNA‐Based Intervention in Oral Submucous Fibrosis: A Scoping Review
Source: Int J Dent. 2026 Aug 27;2026:4230936. doi: 10.1155/ijod/4230936 (PMC13519956; doi:10.1155/ijod/4230936)
Supplement: Supplementary file 2 [file IJOD-2026-4230936-s001.docx]

**Table S2.** Database-specific search strategies used for the literature search

| **Database** | **Search Strategies** | **Results** |
| --- | --- | --- |
| PubMed | ((((((oral submucous fibrosis[MeSH Terms]) OR (OSF[Title/Abstract])) OR (OSMF[Title/Abstract])) OR ("oral potentially malignant disorder"[Title/Abstract])) OR ("oral submucosal fibrosis"[Title/Abstract])) AND (((((microrna[MeSH Terms]) OR (MiRNA*[Title/Abstract])) OR (miR[Title/Abstract])) OR ("antifibrotic miRNA"[Title/Abstract])) OR ("antifibrotic miRNA"[Title/Abstract]))) AND ((intervention[Title/Abstract]) OR (therapy[Title/Abstract])) | 11 |
| Embase | ('oral submucous fibrosis'/exp OR osf OR osmf OR 'oral potentially malignant disorders*' OR 'oral submucous fibroses' OR 'oral submucosal fibrosis' OR 'submucous fibrosis' OR 'oral mucosal fibrosis') AND ('microrna'/exp OR mirna* OR microrna* OR mir* OR 'antifibrotic mirna*' OR 'anti-fibrotic mirna*' OR 'anti-fibrotic microrna*' OR 'antifibrotic microrna*') AND (intervention* OR therapy*) | 102 |
| Scopus | ( INDEXTERMS("oral submucous fibrosis") OR TITLE-ABS-KEY(OSF) OR TITLE-ABS-KEY(OSMF) OR TITLE-ABS-KEY("oral potentially malignant disorder*") OR TITLE-ABS-KEY("oral submucosal fibrosis") ) AND ( INDEXTERMS(microrna) OR TITLE-ABS-KEY(miRNA*) OR INDEXTERMS(mirnas) OR TITLE-ABS-KEY("Antifibrotic miRNA*") OR TITLE-ABS-KEY("Anti-fibrotic miRNA*") OR TITLE-ABS-KEY("Anti-fibrotic microRNAs") OR TITLE-ABS-KEY(microRNA*) OR TITLE-ABS-KEY("micro RNA*") ) OR TITLE-ABS-KEY( antifibrotic intervention ) | 155 |
| Web of Science | (((((((("oral submucous fibrosis") OR (OSF)) OR (OSMF)) OR ("fibrosis, oral submucous")) OR ("oral potentially malignant disorders") OR ("oral submucous fibroses")) OR ("oral submucosal fibrosis")) AND (((((((microrna) OR (miRNA)) OR (mirnas)) OR ("Antifibrotic miRNAs")) OR ("Anti-fibrotic miRNAs")) OR (miR”) OR ("Anti-fibrotic microRNAs"))) AND (((intervention) OR (therapy))) | 61 |
